# Enhancement of the cytotoxicity of SR 4233 to normal and malignant tissues by hypoxic breathing.

**DOI:** 10.1038/bjc.1992.409

**Published:** 1992-12

**Authors:** A. I. Minchinton, J. M. Brown

**Affiliations:** Stanford University Medical Center, Department of Radiation Oncology, California 94305-5105.

## Abstract

The bioreductive cytotoxic agent SR 4233 (1,2,4-benzotriazine 3-amine 1,4-dioxide) has been shown to markedly potentiate the cell killing of mouse tumours when combined with fractionated radiation therapy. Differential metabolism under oxic compared to hypoxic conditions results in SR 4233 exhibiting selective cytotoxicity to hypoxic cells. This is thought to result from the production of a cytotoxic free radical which is generated predominantly in the absence of oxygen. We have examined a way of enhancing the effectiveness of this antitumour agent in vivo by artificially increasing the hypoxic fraction of tumours by hypoxic breathing. Mice are placed in a chamber containing 10% Oxygen 90% Nitrogen for 1 h after each administration of SR 4233. Our results in the SCCVII tumour model indicate that this manoeuvre results in a 10-fold increase in antitumour effectiveness of SR 4233 when administered in a fractionated regime with radiotherapy (8 x 2.5 Gy and 0.08 mmol kg-1), but not when a single treatment regime (1 x 20 Gy and 0.3 mmol kg-1) is used. Mathematical modelling of this effect is used to illustrate this phenomenon and can be used to predict the dependence of this type of therapy on the modification of tumour oxygenation.


*Br. J. Cancer* (1992), **66**, 1053–1058 © Macmillan Press Ltd., 1992

### Enhancement of the cytotoxicity of SR 4233 to normal and malignant tissues by hypoxic breathing

A.I. Minchinton & J.M. Brown

*Stanford University Medical Center, Division of Radiation Biology, Department of Radiation Oncology, Stanford, California 94305-5105, USA.*

**Summary** The bioreductive cytotoxic agent SR 4233 (1,2,4-benzotriazine 3-amine 1,4-dioxide) has been shown to markedly potentiate the cell killing of mouse tumours when combined with fractionated radiation therapy. Differential metabolism under oxic compared to hypoxic conditions results in SR 4233 exhibiting selective cytotoxicity to hypoxic cells. This is thought to result from the production of a cytotoxic free radical which is generated predominantly in the absence of oxygen. We have examined a way of enhancing the effectiveness of this antitumour agent *in vivo* by artificially increasing the hypoxic fraction of tumours by hypoxic breathing. Mice are placed in a chamber containing 10% Oxygen 90% Nitrogen for 1 h after each administration of SR 4233. Our results in the SCCVII tumour model indicate that this manoeuvre results in a 10-fold increase in antitumour effectiveness of SR 4233 when administered in a fractionated regime with radiotherapy ($8 \times 2.5$ Gy and 0.08 mmol $kg^{-1}$), but not when a single treatment regime ($1 \times 20$ Gy and 0.3 mmol $kg^{-1}$) is used. Mathematical modelling of this effect is used to illustrate this phenomenon and can be used to predict the dependence of this type of therapy on the modification of tumour oxygenation.

For several decades hypoxic cells residing within tumours have been regarded as a major obstacle to the effectiveness of radiation therapy (Bush *et al.*, 1978; Dische *et al.*, 1983; Henk, 1986). These same cells have also been suggested to represent a population resistant to some chemotherapeutic agents (Kennedy *et al.*, 1980; Tannock & Guttman, 1981; Teicher *et al.*, 1990). Efforts have therefore been made to 'overcome' the resistance of hypoxic cells to radiation using a variety of techniques including treatment with hyperbaric oxygen (Churchill-Davidson *et al.*, 1957; Sealy, 1991; Watson *et al.*, 1978), high LET radiation's (Withers, 1973) and most recently, chemical hypoxic cell radiosensitisers (Adams *et al.*, 1976; Coleman, 1988; Dische, 1991). Hypoxic cell radiosensitisers have, so far, shown only marginal benefit in clinical settings primarily because of dose limiting toxicity (Coleman, 1988; Dische, 1991). However, the quest for improved hypoxic cell radiosensitisers revealed a new class of compounds which exhibit selective toxicity towards hypoxic compared to oxygenated cells (Zeman *et al.*, 1986). SR 4233 (1,2,4-benzotriazine 3-amine 1,4-dioxide) is a lead compound of this new class of antitumour agents and is presently undergoing Phase I clinical trials. The mechanism underlying the selective toxicity of SR 4233 toward hypoxic cells is believed to be due to the activation of SR 4233 by enzyme mediated single electron transfer to a cytotoxic free radical (Baker *et al.*, 1988). When oxygen is present this radical is 'back oxidised' to the parent species possibly allowing other reductive pathways, which do not result in free radical production and hence cytotoxicity, to predominate thus ameliorating the toxicity to oxygenated cells (Baker *et al.*, 1988; Zeman *et al.*, 1986).

The differential cytotoxicity of SR 4233 to oxic and hypoxic cells can be exploited by using radiation to sterilise oxygenated cells and a bioreductive cytotoxin such as SR 4233 to kill the hypoxic cells of tumours. When these two modalities are combined into a fractionated treatment regime (a combination we have termed 'bioreductive radiotherapy') a potent synergistic antitumour effect is obtained (Brown & Lemmon, 1990). The combination of hypoxic cell cytotoxins with radiation given in this form is predicted to be significantly better at controlling tumours than even the most potent hypoxic cell radiosensitiser or fully oxygenating all tumour cells (Brown & Koong, 1991).

Since the oxygen concentration in tumours plays such a crucial role in the bioactivation of SR 4233 to the cytotoxic free radical intermediate, we have examined ways in which the antitumour activity of SR 4233 can be modulated. We exposed tumour bearing mice to a reduced oxygen atmosphere (10% oxygen, 90% nitrogen) for a period of 1 h after treatment with radiation and SR 4233 and tested the effect of this manoeuvre on both single and fractionated treatments *in vivo* using a clonogenic assay. Our data show that the effect of breathing 10% oxygen after radiation is to considerably enhance the antitumour effect of SR 4233, but this only occurs with fractionated, not single dose treatment, radiation treatment. Mathematical modelling indicate that this result is expected provided there is complete reoxygenation and (its counterpart for hypoxic cells, 'rehypoxiation') between treatments.

### Materials and methods

### *Mice and tumour*

Male and female C3H/Km mice were bred and housed under defined flora conditions in the Stanford Research Animal Facility. Food (Wayne autoclavable rodent blox 8656, Madison, WN) and water were available *ad libitum* except for 1 h directly after treatment. The tumours used in this study were SCCVII carcinomas (Hirst & Wood, 1982) intradermally implanted onto the lower back of the mouse and treated when they reached a geometric mean diameter of $8.2 \pm 1.2$ mm, approximately 17 days after implantation of $2 \times 10^5$ cells.

### *Irradiation*

Mice were placed in lead shielding jigs which allowed the tumour and a minimal amount of normal tissue to be irradiated. The radiation; 250 kVp X-rays was delivered using a Phillips RT 250 machine operating at 12.5 mA at a dose rate of ca. 1.8 Gy $min^{-1}$ filtered with 0.35 mm Cu and having a half-value layer of 1.3 mm Cu.

### *Reagents*

SR 4233 was supplied by Dr Michael Tracy (SRI International, Menlo Park, CA) and dissolved in physiological saline

Correspondence: A.I. Minchinton, B.C. Cancer Research Centre, 601 West 10th Avenue, Vancouver, B.C., V5Z 1L3, Canada.
Received 17 January 1992; and in revised form 21 July 1992.

at a concentration of 4 and 6 mmol $dm^{-3}$ respectively for the fractionated and single dose treatments. DNAse and Collagenase were obtained from Sigma St. Louis and Pronase from Calbiochem, San Diego, CA. The doses of SR 4233 were chosen to represent equal proportions of the maximum tolerated dose for single amd multiple administration.

*Treatment protocols*

The single treatment comprised of animals receiving a dose of 20 Gy followed immediately by a dose of 0.3 mmol $kg^{-1}$ SR 4233 i.p. The mice were then placed in a clear plastic box (14 × 14 × 30 cm) which was gassed with 10% oxygen, 90% nitrogen at a flow rate of 5 l $min^{-1}$. The mice remained in this environment for 1 h after which they were returned to normal cages under ambient conditions. The fractionated treatments comprised 8 fractions of radiation, drug and low oxygen breathing. Mice were administered 0.08 mmol $kg^{-1}$ SR 4233 i.p. immediately after receiving 2.5 Gy radiation and preceding 1 h of breathing 10% oxygen 90% nitrogen. Treatments commenced at 8:30 am and 5:00 pm for four consecutive days. Control animals were untreated other than receiving saline injections instead of drug.

*Excision assay*

One day following the end of treatment the mice were killed and tumour response assessed. To do this the tumours wee aseptically removed and weighed, minced with curved scissors and added to 10 ml of Hank's buffered saline solution (Gibco, Grand Island, NY) containing 0.6 mg $ml^{-1}$ Pronase, 0.2 mg $ml^{-1}$ DNAse and 0.2 mg $ml^{-1}$ Collagenase. This mixture was agitated while being maintained at 37°C for 30 min after which the cells were filtered through a steel mesh (pore size approx 100 μm) and centrifuged at 450 × *g* for 10 min. The cell pellet was resuspended in 10 ml of Waymouths medium (Gibco) containing 15% fetal calf serum (Irvine Scientific, Irvine, CA). Cells excluding trypan blue were counted and appropriate dilutions were made prior to plating. The plated cells were then incubated at 37°C for 13 days in humidified air containing 5% $CO_2$.

*Assessment of response*

The action of SR 4233 in combination with radiation on tumours was assessed using the *in vivo - in vitro* excision assay. The number of trypan blue excluding cells extracted per tumour following excision and dissociation was compared to that from control animals in order to obtain an estimate of the fractional yield (FY) of cells from the tumours (i.e. total number of cells recovered from the treated tumour divided by the total number of cells recovered from the control tumours). The surviving fraction of the apparently viable cells extracted from the tumour; i.e. the fraction of cells which go on to form colonies, is calculated by dividing the plating efficiency of the cells from treated animals by the plating efficiency of the control population. By multiplying the surviving fraction by the FY the relative clonogenic cells per tumour is calculated. By examining the three parameters, not only the ultimate outcome of treatment can be observed (the relative clonogenic cells per tumour), but the contribution of the effects on the yield of cells from the tumour as well as the reduction in clonogenicity of those cells can be assessed.

*Toxicity studies*

To assess the effect of breathing 10% oxygen on the toxicity of SR 4233 non-tumour bearing mice were administered SR 4233 either as a single dose or fractionated doses in the same way as the experiments detailed previously. Three mice per dose group were used and the animals were closely monitored for toxicity for up to 30 days after administration. Animals were euthanised if found to be moribund or in distress.

*Assessment of the hypoxic fraction*

The radiobiological hypoxic fraction was determined by comparing the survival of tumour cells irradiated in air breathing animals with the survival of tumour cells made artificially hypoxic by clamping (Van Putten & Kallman, 1968). Clamping was performed for approximately 5 min prior to the irradiation period. The tumours were excised after the irradiation and cell survival assessed by clonogenicity *in vitro*. Two separate experiments were performed each with three mice per dose group.

### Results

*Single treatment protocol*

The pooled results of four experiments performed on male and female mice are shown in Figure 1. Panel a shows values of the relative clonogenic cells per tumour for the eight different treatments. In the unirradiated groups (open symbols) only the treatment involving both drug administration and breathing of 10% oxygen resulted in a decreased value. In the irradiated groups, the fraction of clonogenic cells was reduced to about $10^{-3}$ in animals treated in air without SR 4233 and no difference was seen in the group of animals which breathed 10% oxygen after the irradiation. Tumours from animals receiving SR 4233 and breathing air had a 10-fold reduction in their number of clonogenic cells, but no difference was noted between this group and the animals which breathed 10% oxygen after the irradiation/drug treatment. Panel b of Figure 1 shows the fractional yield of trypan blue excluding cells extracted from the tumours. Tumours from untreated control animals yielded a mean and standard error of $6.06 \pm 1.44 \times 10^7$ cells per tumour. None of the treatments changed the yields of cells from the tumours. Panel c of Figure 1 shows the surviving fraction of the cells extracted from the tumours after different treatments. The unirradiated tumours from mice breathing air or 10% oxygen with or without administration of SR 4233 had similar mean survival. The tumours from mice breathing air or 10% oxygen after radiation treatment had a survival of about $2 \times 10^{-3}$, but the survival of cells from tumours of mice treated with SR 4233 was reduced by about a logarithm to $2 \times 10^{-4}$. Additional treatment with 10% oxygen after irradiation and SR 4233 resulted in no further decrease in survival.

*Fractionated treatment protocol*

The results of experiments performed using a fractionated protocol are shown in Figure 2. Eight doses of 2.5 Gy with or without SR 4233 (0.08 mmol $kg^{-1}$ per inj) were given over 4 days and followed the subsequent day by an excision assay. Panel a shows the relative number of clonogenic cells per tumour resulting from the different treatments. In the unirradiated groups only the tumours from mice treated with both SR 4233 and breathing 10% oxygen had values reduced from treatments without SR 4233. In the irradiated groups of animals which subsequently breathed air or 10% oxygen the number of clonogenic cells was reduced to about $2.5 \times 10^{-3}$. In mice treated with SR 4233 the clonogenic cell survival was further reduced by about one logarithm to $2.5 \times 10^{-4}$, and further reduced to about $2.5 \times 10^{-5}$ when 10% oxygen was breathed in addition to the administration of SR 4233. By examining panel b of Figure 2 it can be seen that the yield of cells was only sizably affected in unirradiated groups by the treatment with both SR 4233 and hypoxic breathing. The apparent decrease in the fractional yield of cells of the irradiated groups shown in panel b occurs because of the effect of the fractionated irradiation on tumour growth, because the tumour growth is retarded by the radiation treatment and therefore the fractional yields are lower than in the unirradiated groups. In the irradiated groups there is a decrease in fractional yield of tumours from mice treated with both SR 4233 and hypoxic breathing. Panel c shows the

**Figure 1** The response of tumours to a single treatment protocol comprising an X-ray dose of 20 Gy and an administration of SR 4233 (0.3 mmol $kg^{-1}$ i.p.). Open symbols represent the results from unirradiated tumours and closed symbols represent irradiated tumours. Results are from four separate experiments, two in female mice, two in males. Each treatment group in each experiment was comprised of four animals. Bars represent ± standard errors.

**Figure 2** The tumour response to a fractionated treatment protocol comprising eight fractions of 2.5 Gy X-rays followed by 0.08 mmol $kg^{-1}$ SR 4233 administration. Open symbols represent the results from unirradiated tumours and closed symbols represent irradiated tumours. Results are from four separate experiments, two in female mice, two in males. Each treatment group in each experiment was comprised of four animals. Bars represent ± standard errors.

surviving fraction of the different treatment groups. All of the unirradiated groups exhibited similar survival. Cells from tumours of mice breathing air or 10% oxygen after the irradiation showed similar survival of approximately $3 \times 10^{-2}$. Tumours from mice receiving SR 4233 had a reduced survival to about $5 \times 10^{-3}$ and this was further reduced by hypoxic breathing to about $1 \times 10^{-3}$.

*Toxicity studies*

The toxicity of the different treatment protocols is shown in Figure 3. The maximum tolerated (MTD) dose for mice breathing air after receiving a single dose of SR 4233 was 0.45 mmol $kg^{-1}$ and for mice breathing the low oxygen mixture the MTD was reduced to 0.2 mmol $kg^{-1}$. In the fractionated studies the maximum tolerated total dose for animals breathing air after receiving an SR 4233 administration was 1.28 mmol $kg^{-1}$ whereas when the animals breathed lowered oxygen this was reduced to 0.8 mmol $kg^{-1}$. Therefore in the single dose studies SR 4233 was 2.25 times more toxic to mice breathing 10% oxygen than air and in the fractionated studies SR 4233 was 1.6 times more toxic to the animals breathing reduced oxygen compared to air breathing.

*Hypoxic fraction determination*

Figure 4 shows the results of two experiments assessing the surviving fraction of tumour cells after graded doses of radiation where the mice were either air breathing of where the tumours were clamped to induce total anoxia. The hypoxic fraction was determined from the separation of terminal slopes of the anoxic and oxic curves. Using this technique

**Figure 3** Toxicity of SR 4233 after single and multiple administration. Mice were administered SR 4233 in an identical fashion to that used in the unirradiated treatment schedules and observed for 30 days. Open and closed circles represent mice receiving a single dose of SR 4233 followed by normal air breathing of 1 h or breathing 10% oxygen 90% nitrogen respectively. Open and closed squares represent mice administered multiple doses of SR 4233 to air breathing or mice breathing 10% oxygen 90% nitrogen for 1 h after each dosing.

**Figure 4** Determination of the radiobiological hypoxic fraction was performed by assessing the survival of cells derived from tumours irradiated in air breathing mice (open symbols) compared to the survival of cells from clamped tumours (closed symbols). The separation of the terminal slopes indicated the fraction of radiobiologically hypoxic cells as described by Moulder and Rockwell, 1984 and is equal to 6.5% (95% confidence limits of 2.82–14.79).

and fitting the data using the method described by Moulder & Rockwell (1984) the tumours were found to have a radiobiological hypoxic fraction of 6.5% (95% confidence limits 2.82–14.76).

### Discussion

The cytotoxicity of SR 4233 is oxygen dependent. *In vitro* studies show that SR 4233 is between 40–150 times more toxic (defined by dose ratios to produce equivalent cytotoxicity) to cells in culture when exposed to the drug under hypoxic compared to oxygenated conditions (Zeman *et al.*, 1986). In the present animal studies we show that in fractionated therapy the anti-tumour effectiveness of SR 4233 can be enhanced by reducing the availability of oxygen post irradiation. However, when a single treatment with radiation and SR 4233 is given no such enhancement in antitumour effectiveness is observed.

The toxicity studies show that hypoxic breathing causes an increase in toxicity in both single and fractionated treatment regimes. In the single dose studies where hypoxic breathing caused no increase in antitumour effectiveness, but caused more toxicity it is clear that no therapeutic advantage is indicated. In the case of the fractionated treatment the increase in toxicity is countered by an increase in antitumour effectiveness. Although full dose response curves would be necessary to quantify the therapeutiv index, it appears that a therapeutic advantage might be obtained with fractionated therapy.

To determine whether the experimental data fit with predictions on the interaction of radiation with a hypoxic cytotoxin, we have modelled each situation. To do this we have assumed that the total tumour cell population can be regarded as being composed of two sub-populations each with a different sensitivity to radiation and an overlapping sub-set of cells that are sensitive to SR 4233. The mathematical models are explained in the Appendix. For the purpose of this discussion we have taken the hypoxic fraction as equal to 5%, close to the actual hypoxic fraction determined in this study, the results of which are shown in Figure 4.

One important finding from the modelling studies is that the model cannot account for the experimental data unless the fraction of cells that is killed by the bioreactive drug is greater than the fraction of cells that are radiobiologically hypoxic. This is in agreement with *in vitro* studies of the oxygen dependence of cell killing by SR 4233 (Koch, C.J., personal communication, 1991 and Tosto & Brown, unpublished). A separate function has therefore been invoked which represents the fraction of the population that is sensitive to the killing effect of the bioreductive drug. We have called this fraction the chemosensitive hypoxic fraction (CHF or $\psi$). Figure 5 shows the overall surviving fraction when 20 Gy radiation and a bioreductive agent (such as SR 4233) is administered as a single fraction. If no drug is administered the survival is approximately $10^{-3}$ which corresponds to the experimental results seen in Figure 1. Experimentally, when SR 4233 is administered, as in Figure 1, this survival is reduced 10-fold. In order to obtain this reduction in survival using the model shown in Figure 5 at least half the population of the tumour must be sensitive to the bioreductive agent, i.e. $\psi$ must equal 0.5, and approximately 95% of the chemosensitive cells must be killed by the administration of the drug. The model then predicts that increasing the proportion of chemosensitive cells would increase the cell kill only marginally and this agrees with the experimental data shown in Figure 1.

For fractionated irradiation with drug treatment the model predicts a different result. Figure 6 shows the effect on surviving fraction of the killing of different proportions of hypoxic cells by the drug on tumours containing different hypoxic fractions. When no killing is achieved by the drug the survival is approximately $10^{-3}$, which corresponds approximately to the survival seen experimentally and shown in Figure 2 for tumours receiving 8 × 2.5 Gy alone. The survival after drug treatment in air breathing animals is reduced about 10-fold. To model this assuming $\psi$ equals 0.5 (as was the case in the single dose studies), then the drug must kill about 50% of the cells (SFd = 0.5). The proportion of cells killed by each dose in the fractionated treatment would be expected to be smaller than that in the single treatment because of the lower drug dose administered: 0.08 mmol kg$^{-1}$ *vs* 0.3 mmol kg$^{-1}$. The model then predicts that doubling the hypoxia causes the survival to be further reduced by about another 10-fold and this is consistent with

**Figure 5** The modelled relationship between overall surviving fraction and the proportion of cells killed by a bioreductive drug for a single dose of 20 Gy X-rays and a single administration of a bioreductive agent such as SR 4233. The model shows the effect of different chemosensitive fractions (Ψ) from 0 to 1.0.

**Figure 6** The modelled relationship between overall surviving fraction and the proportion of cells killed by a bioreductive drug for a fractionated treatment comprising eight fractions of 2.5 Gy X-rays and a bioreductive agent such as SR 4233. The model shows the effect of different chemosensitive fractions (Ψ) from 0 to 1.0.

the experimental results shown in Figure 2.

Although several assumptions are involved in the modelling of the effect of SR 4233 and hypoxic breathing on the sensitivity of tumours to bioreductive radiotherapy, the procedure provides a useful way of predicting the effect of various physiological and pharmacological manoeuvres. For example comparing Figures 5 and 6 it can be seen that if a high proportion of hypoxic cells are killed by the administration of SR 4233 then a much increased effect is achieved in fractionated compared to single treatment protocols. Also, increasing the proportion of hypoxic cells by hypoxic breathing has little effect on a single treatment, but can considerably enhance the antitumour effectiveness of fractionated protocols.

### Conclusions

The antitumour effectiveness of SR 4233 can be enhanced by increasing tumour hypoxia via exposure to a reduced oxygen environment. This manoeuvre causes some increase in systemic toxicity which may balance part of the improved antitumour effect, but these experiments clearly suggest that changing tumour oxygenation can modulate the therapeutic effect. Since the oxygenation of tumour is unstable and subject to fluctuations this way of improving the effectiveness of bioreductive agents may be a promising avenue of investigation. Modelling of the effectiveness of SR 4233 and hypoxic breathing is a useful means of understanding the most profitable ways of optimising its use.

### References

ADAMS, G.E., FLOCKHART, I.R., SMITHEN, C.E., STRATFORD, I.J., WARDMAN, P. & WATTS, M.E. (1976). Electron-affinic sensitization VII. A correlation between structures and one election reduction potentials and efficiencies of nitroimidazoles as hypoxic cell radiosensitizers. *Radiat. Res.*, **67**, 9-20.

BAKER, M.A., ZEMAN, E.M., HIRST, V.K. & BROWN, J.M. (1988). Metabolism of SR-4233 by Chinese hamster ovary cells: basis of selective cytotoxicity. *Cancer Res.*, **48**, 5947-5952.

BROWN, J.M. & KOONG, A. (1991). The therapeutic advantage of hypoxic cells in tumors: a theoretical study. *J. Natl Cancer Inst.*, **83**, 178-185.

BROWN, J.M. & LEMMON, M.J. (1990). Potentiation by the hypoxic cytotoxin SR 4233 of cell killing produced by fractionated irradiation of mouse tumors. *Cancer Res.*, **50**, 7745-7749.

BUSH, R.S., JENKIN, R.D.T., ALLT, W.E.C., BEALE, F.A., BEAN, H., DEMBO, A.J. & PRINGLE, J.F. (1978). Definitive evidence for hypoxic cells influencing cure in cancer therapy. *Br. J. Cancer*, **37** (suppl III), 302-306.

CHURCHILL-DAVIDSON, I., SANGER, C. & THOMLINSON, R.H. (1957). Oxygenation in radiotherapy II. Clinical application. *Br. J. Radiol.*, **30**, 406-422.

COLEMAN, C.N. (1988). Hypoxia in tumour: a paradigm for the approach to biochemical and physiological heterogeneity. *J. Natl Can. Inst.*, **80**, 310-317.

DISCHE, S. (1991). A review of hypoxic cell radiosensitization. *Int. J. Radiat. Oncol. Biol. Phys.*, **20**, 147-152.

DISCHE, S., ANDERSON, P.J., SEALY, R. & WATSON, E.R. (1983). Carcinoma of the cervix-anaemia, radiotherapy and hyperbaric oxygen supports the importance of hypoxia in radiotherapy. *Br. J. Radiol.*, **56**, 251-255.

HENK, J.M. (1986). Late results of a trial of hyperbaric oxygen and radiotherapy in head and neck cancer: a rationale for hypoxic cell radiosensitizers. *Int. J. Radiat. Oncol. Biol. Phys.*, **12**, 1339-1341.

HIRST, D.G. & WOOD, P.J. (1982). The adaptive response of mouse tumours to anemia and retransfusion. *Int. J. Radiat. Biol.*, **51**, 597-609.

KENNEDY, K.A., TEICHER, B.A., ROCKWELL, S. & SARTORELLI, A.C. (1980). The hypoxic tumour cell: a target for selective cancer chemotherapy. *Biochem. Pharmacol.*, **29**, 1-8.

MOULDER, J.E. & ROCKWELL, S. (1984). Hypoxic fractions of solid tumors: experimental techniques, methods of analysis, and a survey of existing data. *Int. J. Radiat. Oncol. Biol. Phys.*, **10**, 695-712.

SEALY, R. (1991). Hyperbaric oxygen in the radiation treatment of head and neck cancers. *Radiother. Oncol.*, **Suppl. 20**, 75-79.

TANNOCK, I. & GUTTMAN, P. (1981). Response of chinese hamster ovary cells to anti-cancer drugs under aerobic and hypoxic conditions. *Br. J. Cancer*, **43**, 245-248.

TEICHER, B.A., HOLDEN, S.A., AL-ACHI, A. & HERMAN, T.S. (1990). Classification of antineoplastic treatments by their differential toxicity toward putative oxygenated and hypoxic tumor subpopulations *in vivo* in the FSaIIC murine fibrosarcoma. *Cancer Res.*, **50**, 3339-3344.

VAN PUTTEN, L.M. & KALLMAN, R.F. (1968). Oxygenation status of a transplantable tumor during fractionated radiotherapy. *J. Natl Cancer Inst.*, **40**, 441-451.

WATSON, E.R., HALNAN, K.E., DISCHE, S., SAUNDRES, M.I., CADE, I.S., MCEWAN, J.B., WIERNEIK, G., PERRINS, D.J.D. & SUTHERLAND, I. (1978). Hyperbaric oxygen and radiotherapy. A MRC trial in carcinoma of the cervix. *Br. J. Radio.*, **51**, 879-887.

WITHERS, H.R. (1973). Biological basis for high-LET radiotherapy. *Radiol.*, **108**, 131-137.

ZEMAN, E.M., BROWN, J.M., LEMMON, M.J., HIRST, V.K. & LEE, W.W. (1986). SR-4233: a bioreductive agent with high selective toxicity for hypoxic cells. *Int. J. Radiat. Biol. Phys.*, **12**, 1239-1242.

### Appendix

The overall survival after radiation treatments followed by bioreductive drug treatment can be described by:

$$SF_{overall} = ((SF_a + SF_b) \cdot SF_d)^n$$

where $SF_{overall}$ is the overall survival of the tumour population and $SF_a$ and $SF_b$ represent the survival of the oxic and hypoxic proportions of cells respectively to radiation treatment and $SF_d$ represents the surviving fraction after drug treatment and n is the number of fractions. This survival can be written in more detail as:

$$SF_{overall} = (SF_o \cdot (1 - \Phi) + SF_h \cdot \Phi) \cdot (1 - (\Psi \cdot (1 - SF_d))^n$$

where $SF_o$ and $SF_h$ are the surviving fractions of the radiobiologically oxic and hypoxic populations respectively given by linear quadratic model. $SF_d$ is the surviving fraction of the chemotherapeutic hypoxic fraction, $\Phi$ is the radiobiologically hypoxic fraction, and $\Psi$ the chemotherapeutic hypoxic fraction. The linear quadratic model $SF = e^{-(\alpha D + \beta D^2)}$ is used to calculate the model. A hypoxic fraction of 5% has been assumed in this study and $\alpha$ and $\beta$ constants of the linear quadratic model for oxic and hypoxic cells have been assigned values of 0.3, 0.03 and 0.1, 0.0033 respectively, i.e. an O.E.R. of 3. An assumption made in this model is that complete reoxygenation of this tumour occurs between each fraction.

The authors wish to thank Douglas R. Menke and Nixy Zutshi for their technical assistance. This work was suppoorted by grant CA 25990 from the NCl (USA).


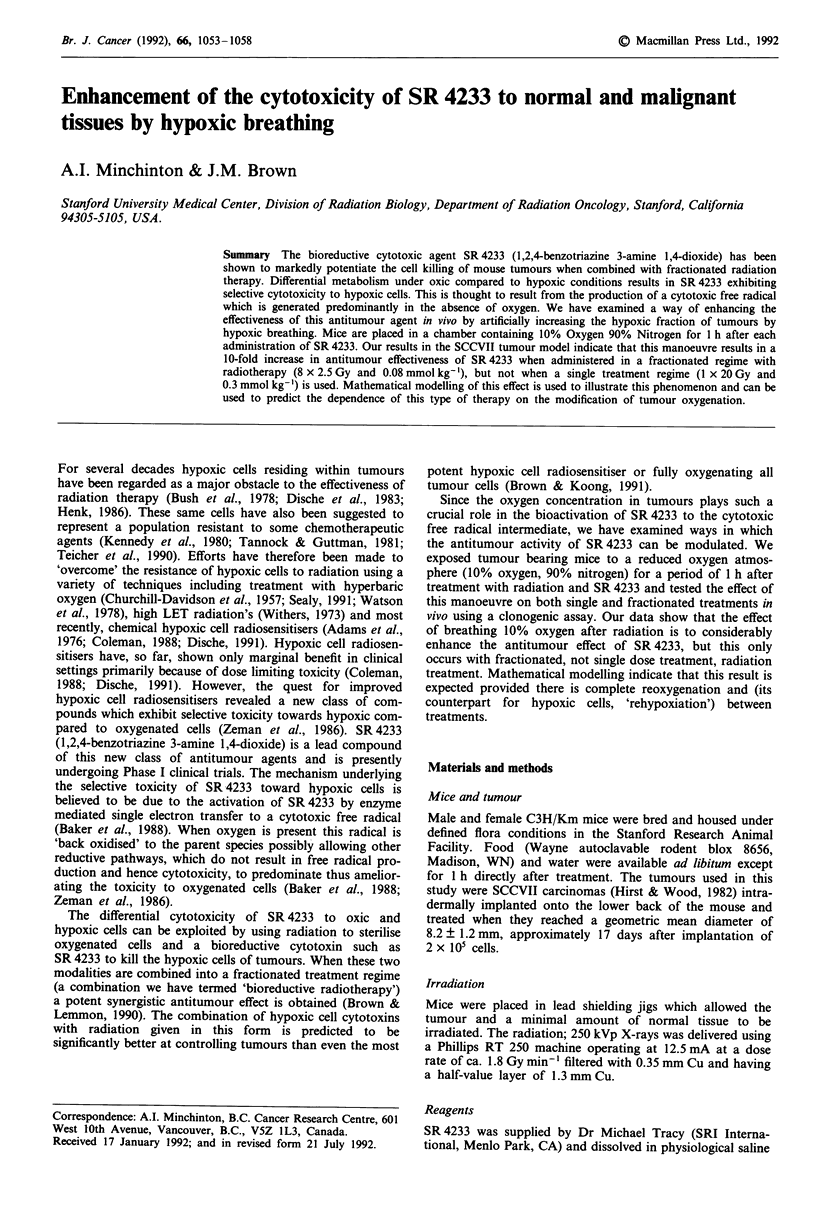

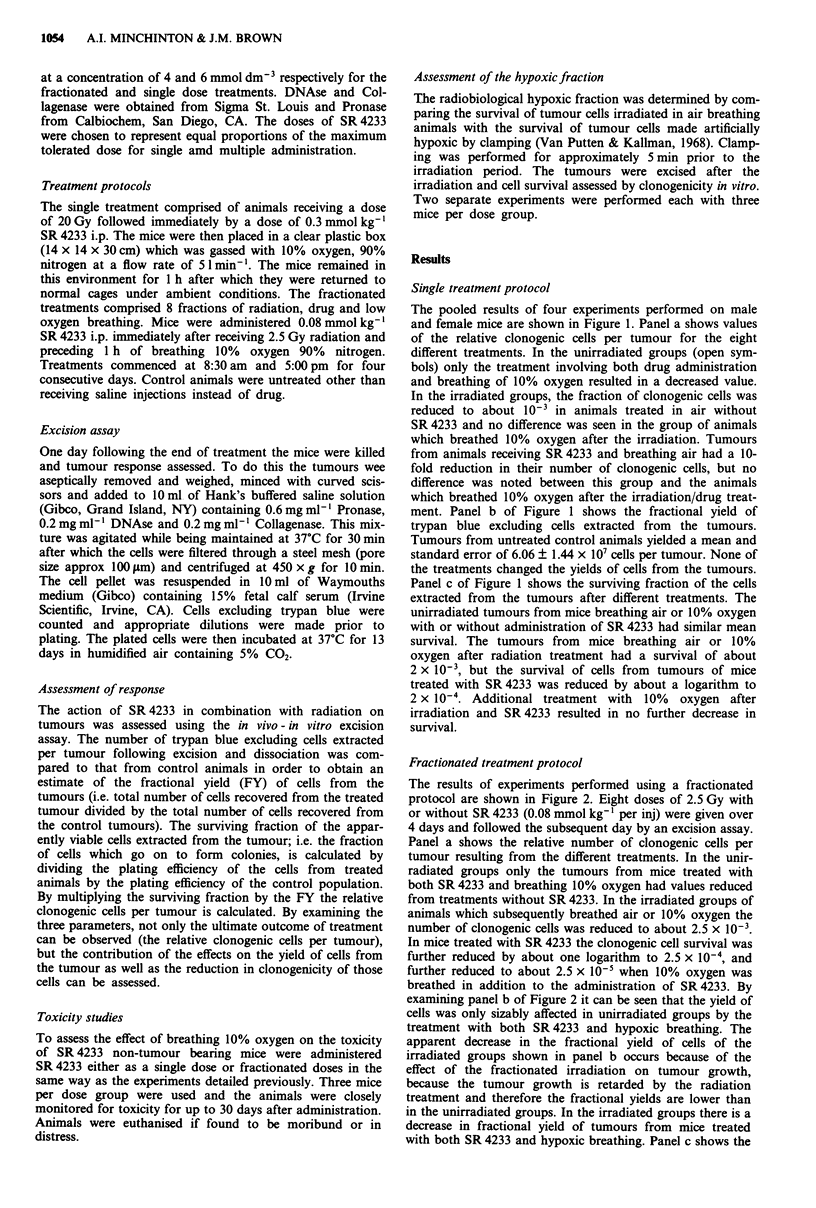

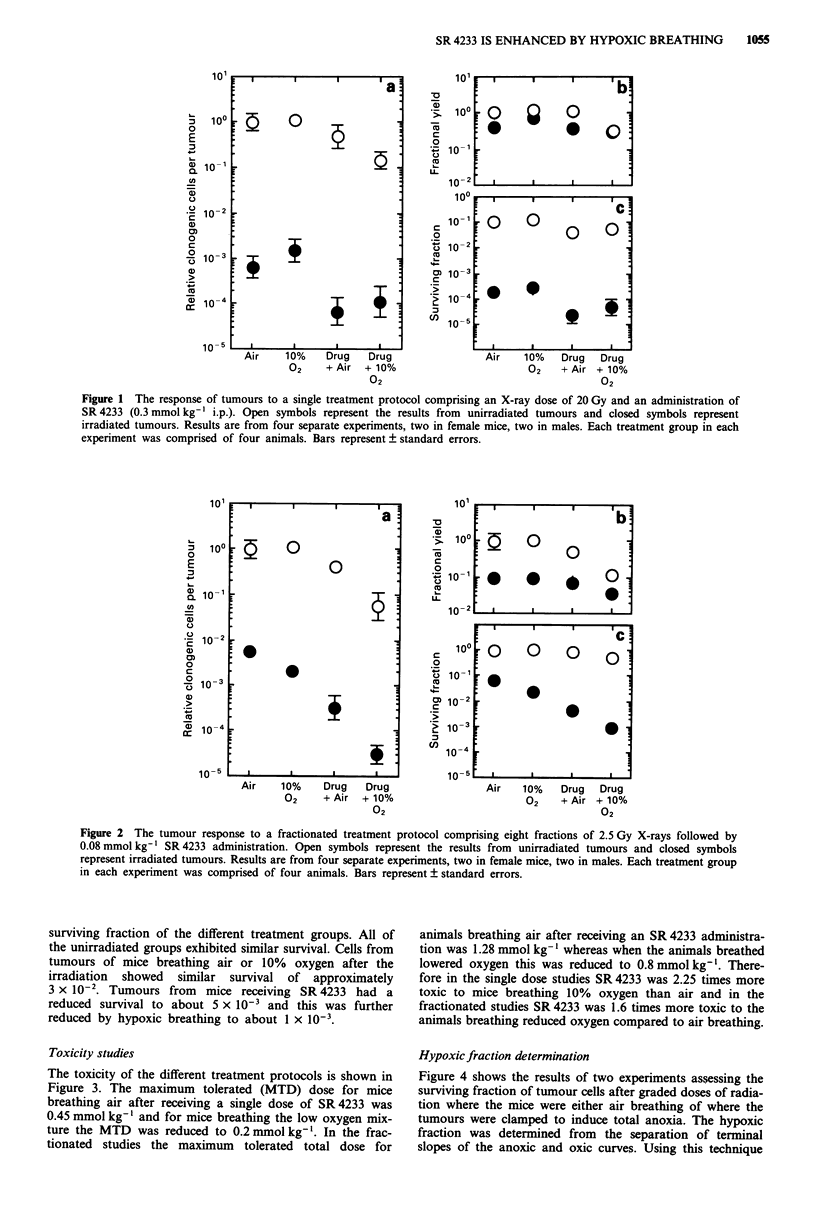

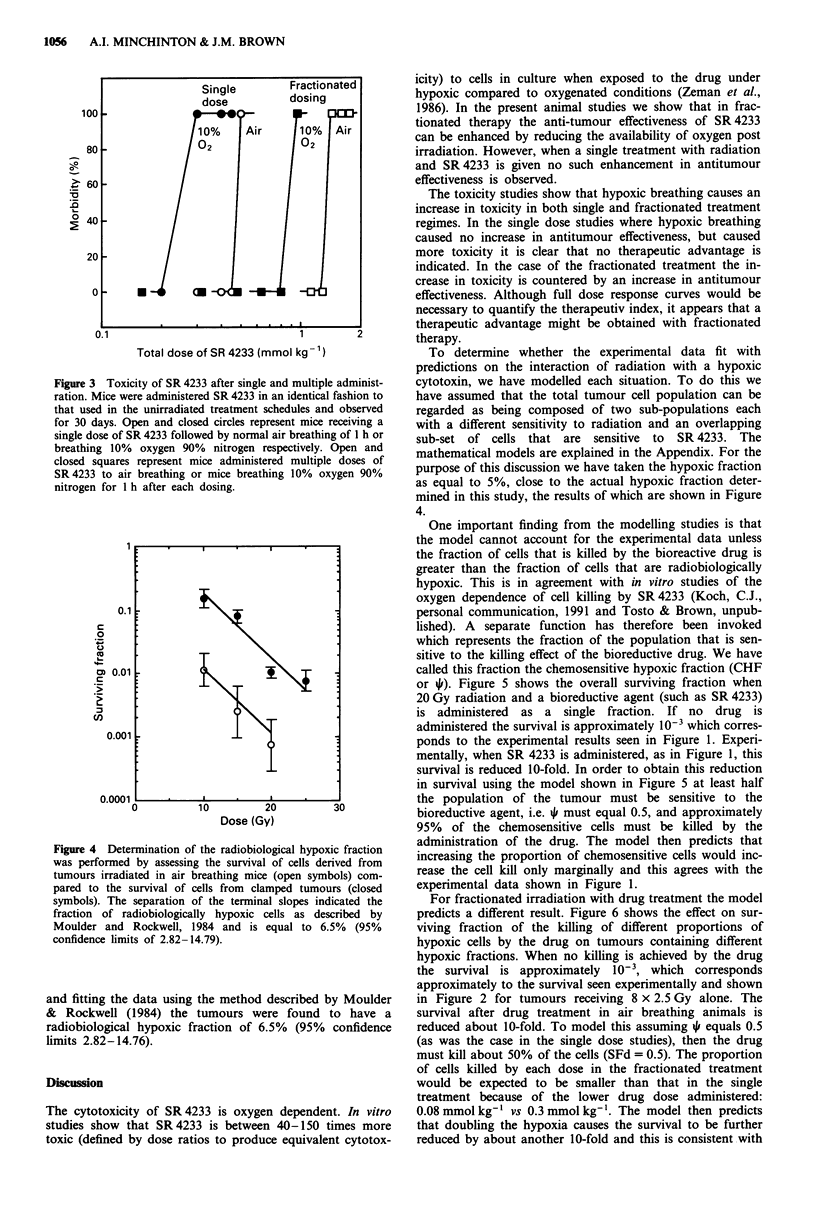

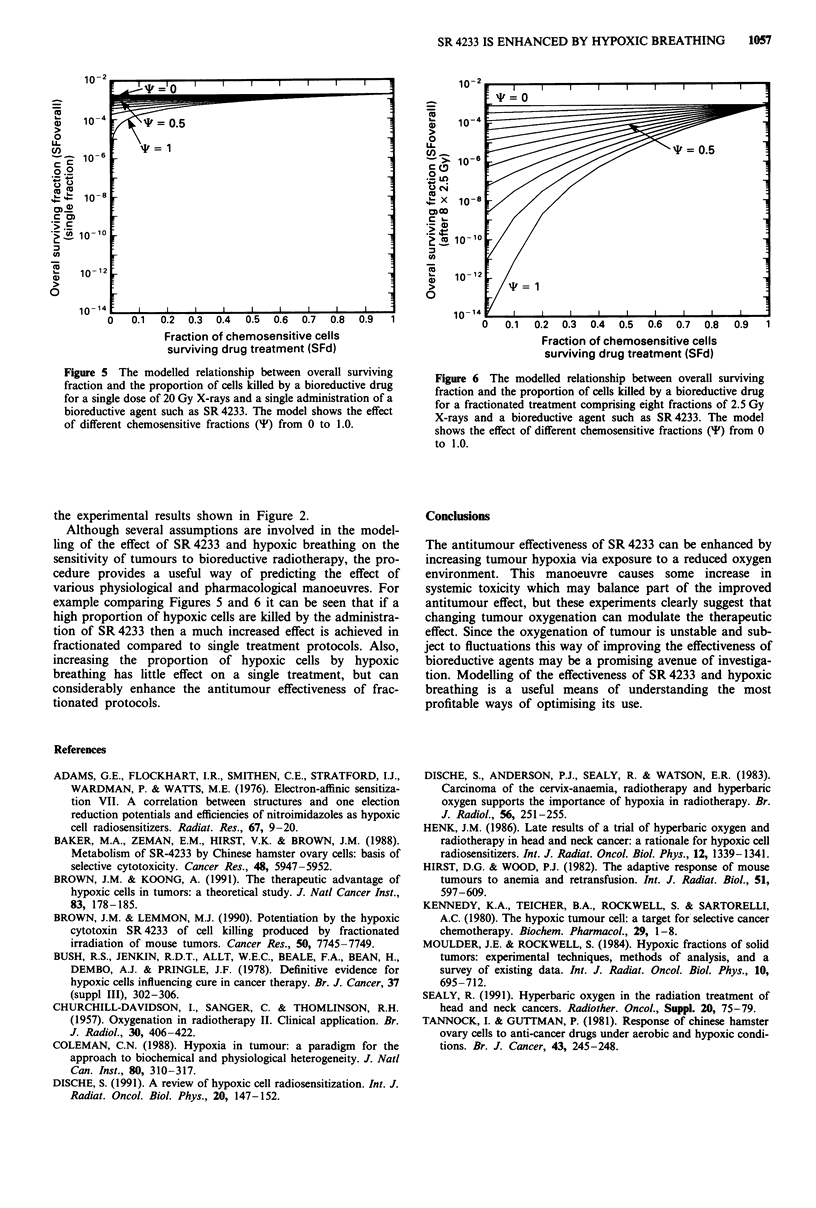

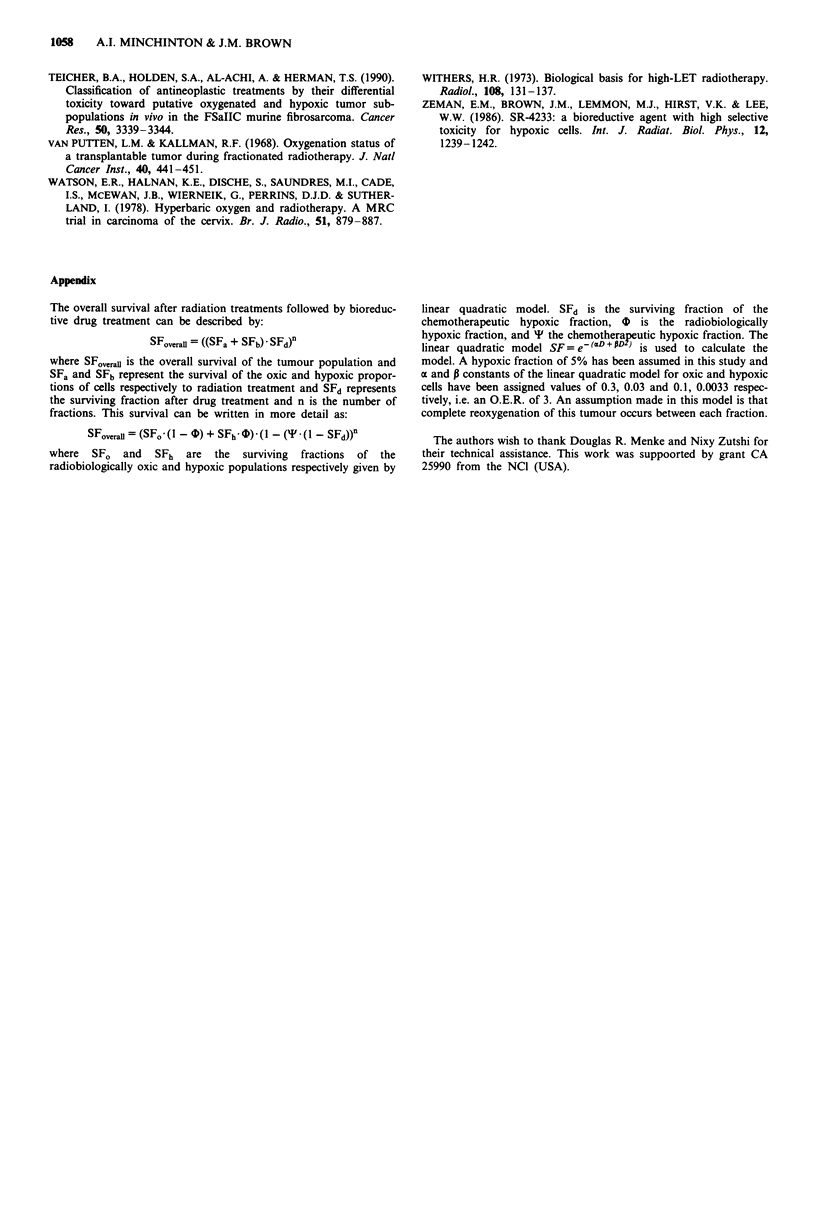


## References

[OCR_00810] Adams G. E., Flockhart I. R., Smithen C. E., Stratford I. J., Wardman P., Watts M. E. (1976). Electron-affinic sensitization. VII. A correlation between structures, one-electron reduction potentials, and efficiencies of nitroimidazoles as hypoxic cell radiosensitizers.. Radiat Res.

[OCR_00817] Baker M. A., Zeman E. M., Hirst V. K., Brown J. M. (1988). Metabolism of SR 4233 by Chinese hamster ovary cells: basis of selective hypoxic cytotoxicity.. Cancer Res.

[OCR_00822] Brown J. M., Koong A. (1991). Therapeutic advantage of hypoxic cells in tumors: a theoretical study.. J Natl Cancer Inst.

[OCR_00827] Brown J. M., Lemmon M. J. (1990). Potentiation by the hypoxic cytotoxin SR 4233 of cell killing produced by fractionated irradiation of mouse tumors.. Cancer Res.

[OCR_00832] Bush R. S., Jenkin R. D., Allt W. E., Beale F. A., Bean H., Dembo A. J., Pringle J. F. (1978). Definitive evidence for hypoxic cells influencing cure in cancer therapy.. Br J Cancer Suppl.

[OCR_00838] CHURCHILL-DAVIDSON I., SANGER C., THOMLINSON R. H. (1957). Oxygenation in radiotherapy. II. Clinical application.. Br J Radiol.

[OCR_00843] Coleman C. N. (1988). Hypoxia in tumors: a paradigm for the approach to biochemical and physiologic heterogeneity.. J Natl Cancer Inst.

[OCR_00848] Dische S. (1991). A review of hypoxic cell radiosensitization.. Int J Radiat Oncol Biol Phys.

[OCR_00852] Dische S., Anderson P. J., Sealy R., Watson E. R. (1983). Carcinoma of the cervix--anaemia, radiotherapy and hyperbaric oxygen.. Br J Radiol.

[OCR_00858] Henk J. M. (1986). Late results of a trial of hyperbaric oxygen and radiotherapy in head and neck cancer: a rationale for hypoxic cell sensitizers?. Int J Radiat Oncol Biol Phys.

[OCR_00862] Hirst D. G., Wood P. J. (1987). The adaptive response of mouse tumours to anaemia and retransfusion.. Int J Radiat Biol Relat Stud Phys Chem Med.

[OCR_00867] Kennedy K. A., Teicher B. A., Rockwell S., Sartorelli A. C. (1980). The hypoxic tumor cell: a target for selective cancer chemotherapy.. Biochem Pharmacol.

[OCR_00872] Moulder J. E., Rockwell S. (1984). Hypoxic fractions of solid tumors: experimental techniques, methods of analysis, and a survey of existing data.. Int J Radiat Oncol Biol Phys.

[OCR_00878] Sealy R. (1991). Hyperbaric oxygen in the radiation treatment of head and neck cancers.. Radiother Oncol.

[OCR_00882] Tannock I., Guttman P. (1981). Response of Chinese hamster ovary cells to anticancer drugs under aerobic and hypoxic conditions.. Br J Cancer.

[OCR_00889] Teicher B. A., Holden S. A., al-Achi A., Herman T. S. (1990). Classification of antineoplastic treatments by their differential toxicity toward putative oxygenated and hypoxic tumor subpopulations in vivo in the FSaIIC murine fibrosarcoma.. Cancer Res.

[OCR_00896] Van Putten L. M., Kallman R. F. (1968). Oxygenation status of a transplantable tumor during fractionated radiation therapy.. J Natl Cancer Inst.

[OCR_00907] Withers H. R. (1973). Biological basis for high-LET radiotherapy.. Radiology.

[OCR_00911] Zeman E. M., Brown J. M., Lemmon M. J., Hirst V. K., Lee W. W. (1986). SR-4233: a new bioreductive agent with high selective toxicity for hypoxic mammalian cells.. Int J Radiat Oncol Biol Phys.

